# Seed Thermal Response, and Morphological and Biochemical Plasticity in *Wigandia urens* as Indicators of Its Utility for Elevational and Latitudinal Assisted Migration

**DOI:** 10.1002/ece3.72694

**Published:** 2025-12-25

**Authors:** Ivonne Reyes‐Ortega, María E. Sánchez‐Coronado, Susana Orozco‐Segovia, César M. Flores‐Ortiz, Gastón Contreras‐Jiménez, Marco Solano‐De la Cruz, Sonia Juárez‐Orozco, Zenón Cano‐Santana, Graciela García‐Guzmán, Jorge R. Blanco‐Martínez, Alma Orozco‐Segovia

**Affiliations:** ^1^ Departamento de Ecología Funcional, Instituto de Ecología Universidad Nacional Autónoma de México Ciudad de México México; ^2^ Facultad de Ciencias, División de Estudios de Posgrado Universidad Nacional Autónoma de México Ciudad de México México; ^3^ Departamento de Física, Facultad de Ciencias Universidad Nacional Autónoma de México Ciudad de México México; ^4^ Laboratorio de Fisiología Vegetal, UBIPRO, Facultad de Estudios Superiores Iztacala Universidad Nacional Autónoma de México México México; ^5^ Laboratorio de Microscopía y Microdisección Laser y Electroquímica, Instituto de Ecología Universidad Nacional Autónoma de México Ciudad de México México; ^6^ Unidad de Genética Molecular, Instituto de Ecología Universidad Nacional Autónoma de México Ciudad de México México; ^7^ Departamento de Ecología y Recursos Naturales, Facultad de Ciencias Universidad Nacional Autónoma de México Ciudad de México México; ^8^ Secretaría Técnica y de Gestión, Instituto de Ecología Universidad Nacional Autónoma de México Ciudad de México México; ^9^ Departamento de Biología Comparada, Facultad de Ciencia Universidad Nacional Autónoma de México Ciudad de México México

**Keywords:** base and ceiling temperatures, cumulative soil temperature, elevational gradient, maternal effects, stress temperature memory, thermal time model

## Abstract

Thermal time models are useful for predicting the effects of global climate change on plants and enable us to know the phenotypic plasticity of germination responses to temperature. In five populations of *Wigandia urens* collected in Mexico at elevations of 1260, 1660, 2040, 2345, and 2500 m above sea level (a.s.l.), we determined soil temperatures, seed traits, cardinal temperatures, thermal time (*T*_t_), and fatty acid content. In seeds collected at 2345 m, we also assessed the effect of osmotic potential on germination. Temperature in the elevational gradient differed by 10.7°C ± 1.37°C, variation higher than the increase in (6°C) predicted by CC models for the next century. Temperature was negatively related to elevation, positively related to germination capacity, and negatively related to lag time, seed mass, and lipid content. All elevational seed populations exhibited high germination capacity and a wide thermal germination window. Germination was thermophilic. *T*_base_ for germination was 9.81°C–12.48°C and *T*_ceiling_ ≈ 34.74°C. Variation in *T*_t_ was broader than that in *T*_base_ and *T*_ceiling_. Elevation and/or percentile subpopulations significantly affected *T*_base_ and *T*_t_, while elevation affected *T*_ceiling_. For suboptimal and supraoptimal *T*_t_, the lowest values were at 1660 m and the highest at 2345 and 2500 m. Suboptimal *T*_t_ exhibited greater variation than the other parameters, probably determining the *W. urens* distribution. This species germinated from 0 to −0.5 MPa in 2.4 days, the time required to reach a cumulative soil temperature equivalent to *T*_t_ for the 50% percentile. Even 32% germinated at −1 MPa. Unsaturated fatty acids content was highest at 2500 m. The phenotypic plasticity of *W. urens,* expressed in its germination and ecological distribution, suggests that it is a model pioneer tree useful as a facilitator species capable of facing temperature and precipitation predicted by climatic change and beneficial for ecological restoration and/or assisted migration across elevational or latitudinal gradients.

## Graphical abstract

To identify the phenotypic plasticity in germination traits related to the tolerance to changes in temperature resulting from CC and improve the successful of plant cover recovery in elevational and latitudinal gradients, we assessed the amplitude and variability of phenotypic plasticity in the seed response of *W. urens* to temperature, throughout thermal time models, fatty acid profile and their concentrations in an elevational gradient, relating them to soil and air temperatures and seed morphological traits. Based on the results we propose that *W. urens* and the analytical method used in this research are useful to improve projects for restoration and/or for assisted migration in elevational and latitudinal gradients and to identify species with ample phenotypic plasticity.

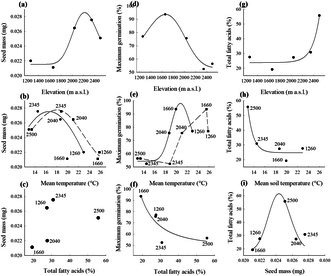

## 1 Introduction

Carbon dioxide and other gas emissions significantly contribute to climate change (CC; Sentinella et al. 2020; Siyum 2020, Forster et al. 2023), impacting temperature and precipitation worldwide (Sanchez‐Azofeifa and Stan 2019; WMO 2019, 2023; Ohba 2021). These two climate elements are fundamental for meeting the temperature and water availability requirements for seed germination, plant growth, and reproduction (Walck et al. 2011; Moles et al. 2014).

From 2013 to 2022, CC induced an increase of 2°C per decade (Forster et al. 2023). Moreover, the International Commission on Climate Change forecasts that if current trends in fossil fuel use and deforestation are not reversed, the mean global temperature could rise by 6°C over the next century (Arnell et al. 2019; Bendou et al. 2022). Due to CC, species may move from lower to higher elevations or latitudes (Vitt et al. 2010), as occurred during the late Pleistocene–Holocene interval when environmental temperatures increased by 6°C (Bush et al. 2004). Currently, it is possible to model temperature around the world, thanks to satellites, Geographic Information Systems, and other tools. But CC's impacts on precipitation are unpredictable across elevation and latitude, and at a global scale, there are few experimental measures to model it (Ooi et al. 2012; Cowles et al. 2018; Pepin et al. 2022).

The current rate of temperature increase is unprecedented, with its effects manifesting in just a few years (Tierney et al. 2020). Consequently, plant plasticity—magnitude and interval of phenological and physiological responses in plants, Richardson et al. 2017—can play a critical role in promoting plant survival and migration (assisted or not) to new distribution areas.

For seed development and germination, temperature parameters and their variations in time and space (at different scales, Stull 1988) define the germination niche of the species (Grubb 1977; Luna et al. 2012), for instance, α and β scales (20 m to 2 km Stull 1988) are decisive for seed development and maturation, while for seeds in the soil bank and during germination, microscale γ and δ (from 2 m to 2 mm) are relevant. Generally, temperatures in elevational gradients vary by 0.6°C for every 100 m (Ooi et al. 2012), which can affect plant growth and reproductive biology (Bykova et al. 2012). This value is not constant and varies globally at different scales, as temperature is closely related to latitude, elevation, topography, and the microtopographic characteristics of each site (La Sorte et al. 2014).

The effects of environmental temperature and water availability during seed development are known as maternal effects, resulting in ecophysiological changes in seeds and progeny due to phenotypic plasticity (Gutterman 2000; Fernández‐Pascual et al. 2019; Kijowska‐Oberc et al. 2020). For instance, depending on environmental conditions, seeds of a species can produce stress proteins, polyunsaturated lipids, antioxidants, etc., enabling them to withstand high temperatures from germination to the following plant phenological stages (González‐Zertuche et al. 2001; Gamboa‐deBuen et al. 2006; Tonguç et al. 2022). Currently, maternal effects on seeds are described as the environmental information that plants acquire from their parents or themselves, inducing a “temperature and water stress memory”. Stress memory implies that a stressful environmental factor induces the acquisition of information (e.g., biochemical) that allows plants to cope with future stressful environmental conditions (Gamboa‐deBuen et al. 2006; Peng et al. 2019; Sharma et al. 2022). In these changes, plant genetic variation is involved (Arnold et al. 2022), although epigenetic changes do not necessarily occur in a short period, as those occurring in CC (Potvin and Tousignant 1996; Merilä and Hendry 2014; Miryeganeh and Armitage 2025). Variation in germinability, due to temperature during seed development and maturation (Dorne 1981), can be tested in temperature gradients, as intraspecific variation in seed germination (germination capacity, cardinal temperatures—optimal, base, and ceiling—and thermal time; Garcia‐Huidobro et al. 1982; Fernández‐Pascual et al. 2019). The rapid temperature changes due to climate change (that may be occurring at the same time that environmental changes are occurring in the changing environment of the earth) might enable plants to acclimatize to new environments through changes that improve adaptability; thus, plasticity may serve as the first line of defense for plants facing CC and the potential to extend their limits due to temperature stress memory, within their phylogenetic constraints (Potvin and Tousignant 1996; Donohue et al. 2010; Merilä and Hendry 2014). Therefore, a clear understanding of the potential responses of physiological traits in plants to anticipated climate changes is essential for predicting trends in shifts or declines in species range, persistence in situ due to local acclimatization, local extirpation, and/or global extinction (McCarty 2001; Walther et al. 2002, 2005; Davis et al. 2005). In this context, maintaining or restoring ecosystem functionality and, consequently, preserving ecosystem services (*sensu* Nápoles‐Vértiz and Caro‐Borrero 2024) are crucial. It is hence necessary to identify species or groups of species that facilitate these processes. For instance, pioneer species, such as *W. urens*, may play an important role due to their adaptation to adverse environmental conditions created by natural disturbances or human‐induced ecosystem deterioration (Vázquez‐Yanes and Orozco‐Segovia 1984; Dalling and Burslem 2008; Gbètoho et al. 2017). They generate new microclimate conditions suitable for establishing species from mature communities. Since the 1970s, plant species distribution has been explained with reference to temperature–time relations in germination (Thompson 1970a, 1970b, 1973, 1975; Linder 2000). Now, thermal time models are an important tool, but are little used to predict the effects of CC on plant species distribution and their potential performance beyond their current distribution areas, aiding in the design of restoration ecology plans, assisted migration, protection of endangered plants, or identification of key species for reintroduction in deforested areas (Root et al. 2003; Duarte et al. 2018; Sampayo‐Maldonado et al. 2019). Based on literature (see e.g., Lira‐Caballero et al. 2018), vegetation recovery, assisted migration, and protection of endangered species might be facilitated across elevational gradients for *W. urens* , a pioneer and engineering species that could create adequate niches for species displaced by CC in a wide temperature gradient.

In this context, our objectives were as follows: (1) To assess the seed response to temperature (seed germination capacity—seeds capable of completing germination in a determined condition—, cardinal temperatures—optimal, base, and ceiling—and thermal time) of five populations of *Wigandia urens* collected along an elevational gradient ranging from 1260 to 2500 m in Central Mexico. (2) To relate seed mass, seed area, and lipid content to elevation, soil and air temperatures, lag time, and seed germination capacity. (3) To determine whether the germinative seed response to temperature in the elevational seed populations of *W. urens* reflects their phenotypic plasticity and the potential temperature memory stress generated by environmental temperatures along the gradient. The experimental temperatures included extreme soil temperatures recorded during the rainy season (5°C–41°C). (4) Since CC effects on precipitation are projected, we assessed the effect of osmotic potential on the germination of a seed population (2345 m a.s.l) growing in an environment with water stress.

## 2 Materials and Methods

### 2.1 Study Species

*Wigandia urens* (Ruiz & Pavón) Kunth (Boraginaceae) is a tropical pioneer tree commonly found in disturbed habitats, widely distributed from Sinaloa, Mexico, to Venezuela, Colombia, and Peru (Ochoa 2001). It has been introduced to Africa, North and South America (Argentina, California), Asia (China), Europe (France, Italy), and Australia; where it has been considered a potential invader but is controlled by the presence of native plants (Chandra et al. 2023; EPPO Global Data Base 2025). This species colonizes disturbed mountain cloud forests, temperate and tropical deciduous forests, xerophilic shrublands (Rzedowski and de Rzedowski 2019), and areas in Central Mexico undergoing ecological restoration in lava fields; however, it has low dominance in advanced stages of restoration (González‐Jaramillo 2018; Z. Cano‐Santana, personal observations). In Central America and Mexico, it grows up to 3600 m a.s.l. (CONABIO 2025; EPPO Global Data Base 2025) and is notable for producing a high amount of organic matter and providing food and shelter for over 25 species of epiphytic arthropods (Cano‐Santana 1987, 1994; Cano‐Santana and Oyama 1994), even in urbanized areas (Z. Cano‐Santana, personal observations). Blooming occurs from November to February or March.

From April to May 1998, seeds were collected (~6 g per site) at three locations along Route 113 (Mexico City–Oaxtepec) and two in Mexico City. These sites were located along an elevational gradient between 1260 and 2500 m a.s.l. The localities along the road were Yautepec (1260 m), Tlayacapan (1660 m), and Tlalnepantla (2040 m) in Morelos State; the Ecological Reserve El Pedregal de San Ángel (REPSA, 2345 m); and the Parque Ecológico de la Ciudad de México (2500 m) in Mexico City. The elevational difference between the populations was 360 ± 41 m. The seeds were stored in paper bags under laboratory conditions (21.6°C ± 1.8°C; RH = 38.7% ± 6.3%). All experiments and measurements were carried out in the same year or 2 years after seed collection. Table 1.

**TABLE 1:** Replication statements for the experimental design examining how seed collection site (elevation m a.s.l.) and a temperature gradient affect the germination response of the *Wigandia urens* seeds.

Scale of inference	Scale at which the factor of interest is applied	Number of replicates at the appropriate scale
**Soil and air temperatures**		
Site type	Seeds collection sites (elevation: 1260, 1660, 2040, 2345 or 2500 m a.s.l.)	5 dataloggers per elevation
**Seed traits**		
Seed mass Seed area Seed number per capsule Capsules in scorpioid cymes	Individuals	15 seeds per elevation 15 capsules 15 scorpioid cymes
	Individuals from 1345 m a.s.l.	
Fatty acids identification and quantification	Oil sample	1 replicate per elevation
**Germination capacity (dormancy)**		
Seed samples sown in 5 different GA_3_ concentrations (0, 500, 1000, 1500, 2000 ppm)	Petri dishes	3 replicates of 50 seeds in each petri dish per elevation
**Effect of osmotic potential on seed germination**		
Seed samples sown in each one of 7 different osmotic potentials (0, −0.5, −1, −1.5, −2, −2.5, −4 MPa)	Petri dishes	5 replicates of 50 seeds in each petri dish tested in seeds from the 2345 elevational population
**Effect of a temperature gradient on seed germination**		
Seed samples sown in each 1 of 15 different temperatures (5°C, 7°C, 9°C, 11°C, 14°C, 17°C, 21°C, 23°C, 25°C, 28°C, 31°C, 33°C, 35°C, 37°C, or 41°C). In analyses were included the nine temperatures (11°C–33°C) where the germination was higher than zero	Test tubes	Per elevation, 5 replicates of 50 seeds in each test tube

Seed collection was carried out as described in https://www.bloomingboulevards.org/ethical‐standards‐forr‐naseed‐collecting and https://plantpropagation.org/seed‐collection‐protocols/. Seed collection permission was obtained from the Secretaría del Medio Ambiente, Recursos Naturales, y Pesca: DOO.02.6540.

### 2.2 Soil and Air Temperatures at the Collection Site

The soil temperature at each seed collection site was measured during the dry season (May). Simultaneously, in each locality along the elevational gradient, a Hobo Temp H01‐001‐01 datalogger (Computer Corporation, Pocasset, MA, USA) was buried for a week at a depth of 3 cm in open sites. The temperature was recorded every 10 min. Differences in temperature between localities were determined using ANOVA and Tukey post hoc tests. We also obtained the air temperature data for May from the nearest meteorological stations located at elevations similar to the collection sites (smn.conagua.gob.mx 2023a, 2023b; observatoriometeorologico.filos.unam.mx 2023; smn.conagua.gob.mx, 2023). The mean, minimum, and maximum temperatures were related to elevation using linear regression.

The rates of change in elevation of minimum, mean, and maximum soil temperatures were calculated from the function:

$$Y=a+b\times x^{3}$$

### 2.3 Seed Traits

#### 2.3.1 Seed Mass, X‐Ray Images and Number of Seeds

Individual seed mass was evaluated in 15 seeds per elevation using a Microbalance Mettler‐Toledo XP6 (Switzerland; readability: 0.0001 mg). We also evaluated the number of seeds per capsule (*n* = 15) and the number of capsules in 15 scorpioid cymes. The presence or absence of empty seeds was evaluated in 15 seeds per elevation using a Cabinet X‐Ray System UltraFocus, Model 451‐B (Faxitron Bioptics LLC, Tucson, AZ, USA).

#### 2.3.2 Lipid Content of the Seeds

To observe lipids' location in the seeds' tissues and determine the seed sample size for the lipid extraction method from each elevation, 5–11 seeds of *W. uren*s (depending on seed availability) were embedded at laboratory temperature in the Tissue‐Tek OCT compound (Sakura Finetek, Torrance, California), in a stainless steel sample holder, and immediately placed on a fast‐freeze stage at −60°C for 3 min. The sample holder was then placed on the specimen head to be sectioned in slices of 20 μm thickness at 22°C in a cryostat (CryoStar NX70, Epredia, London, UK). The slices were immediately transferred to glass slides and dried for 10 min; thereafter, the seed sections were stained with aliquots of 1 mg mL^−1^ in lugol of Oil Red O (Sigma‐Aldrich, USA) for 2 min to stain the lipids. The slides were mounted on an ArcturusXT Nikon Eclipse T*i* microscope (Applied Biosystems, CA, USA), and image (20×) acquisition was carried out in BrightField using the Jenoptik ProgRes c14 Plus CMOS camera in Image Pro Insight software Image Pro Insight v9.3 of MediaCybernetics.

To evaluate the presence and quantity of Linoleic, Oleic, Palmitic, and Stearic acids from each elevational population, 100 mg of seeds were weighed (fresh weight) and frozen with liquid nitrogen and macerated in Qiagen TissueLyser II for 1 min at 30 Hz (180–1800 oscillations/min). Total lipids were extracted using the High Standard Fatty Acid Extraction Kit, Catalog Number MAK338 (Sigma‐Aldrich, Merck KGaA, Darmstadt, Germany), following the manufacturer's specifications. In the extraction solvent solution (3 mL), the macerate was immediately homogenized to avoid thawing and vigorously vortexed for 30 s. We added 0.5 mL of aqueous buffer per sample and vigorously vortexed the sample for 30 s more. The solution obtained was passed through the extraction column, yielding approximately 1 mL of oil, with excess discarded to avoid water residue in the final lipid solution.

The lipid solution was analyzed using a 6850 gas chromatograph and a 5975c mass spectrometer (both, Agilent Technologies, Santa Clara, CA, USA) using a 30 m long capillary column (HP‐5MS, Agilent), 0.25 mm in diameter, and 0.25 μm film. The carrier gas was ultra‐pure helium of ultra‐high purity at a flow rate of 1 mL min^−1^. Independent 2 L samples of 2 μL were injected separately in split mode at 250°C. The gas chromatographer oven was initially set at 150°C for 2 min, and then ramped to 200°C at 5°C min^−1^ for 2 min, followed by a second ramp heated to 240°C at 3°C min^−1^; the total run time was 25.3 min. The temperature was then increased to 225°C at a rate of 1°C min^−1^ and held at this temperature for 5 min. The temperature was further increased to 280°C at a rate of 5°C/min, and then the rate was increased to 20°C min^−1^ until a final temperature of 300°C was reached and held for 3 min. The mass spectrometer was operated in electron impact mode (electron energy = 70 eV), the line ion source temperature was 230°C, and the quadrupole was at 150°C. Mass spectra were recorded under electron ionization (70 eV) within the 35–400 m/z interval. Heptadecanoic acid was used as an internal standard.

Fresh basis of seed water content (WC_fb_) was determined by individually weighing 15 seeds in the Microbalance Mettler‐Toledo, before and after being dried in a mini‐incubator (Labnet International Inc., NJ, USA) at 40°C (at higher temperatures, the seed lipids began to be lost through liquefaction or evaporation, thus seeds are fried and/or burned). Drying continued for a week after seed weight did not change. Thereafter, the WC was calculated afresh as an oil‐free percentage on a fresh basis. Total lipid content (%) on fresh basis = (LC_fb_).

$${\mathrm{WC}}_{\text{offb}}=\frac{\left(100\;x\;{\mathrm{WC}}_{\mathrm{fb}}\right)}{\left(100\;x\;{\mathrm{LC}}_{\mathrm{fb}}\right)}$$

### 2.4 Germination Capacity

The absence of dormancy being a requirement for analyzing germination with thermal time models (Hardegree et al. 1999), we evaluated its presence or absence among elevational seed populations after collection and after 1 and 2 years of storage under laboratory conditions. For all germination tests, three replicates of 50 seeds were sown in Petri dishes (10 cm in diameter) on the surface of plates containing 1% agar in distilled water. The dishes were incubated under light (12 h day^−1^ photoperiod, R:FR = 1.73, PFD = 33.21 μm^−2^ s^−1^ at 25°C) in a germination chamber (Biotronette 844, Lab‐line Instruments Inc., Melrose Park, IL, USA). To test for primary dormancy, 0 (control), 500, 1000, 1500, or 2000 ppm gibberellic acid (GA_3_) was added to the agar. Germination was recorded daily for 1 month, making a total of 450 petri dishes and 2250 seeds.

### 2.5 Determination of the Osmotic Potential in Seed Germination

We determined the effect of the osmotic potential on germination in the seed population growing at 2345 m a.s.l. because in this location soil is sandy‐loam, shallow, and deposited on volcanic rock; it has low water retention and edaphic aridity (García‐Aguirre et al. 2007; Olvera‐Carrillo et al. 2009). Osmotic solutions were prepared using polyethylene glycol 6000 (PEG 6000, Baker, USA) at osmotic potentials of 0 (control), −0.05, −1 MPa, −1.5, −2, −2.5, and −3 MPa. The PEG solutions were prepared according to the software of Michel and Radcliffe (1995). Five replicates of 50 seeds per treatment were sown on the surface of a nylon mesh (to avoid seed oxygen deprivation and allow the free movement of PEG molecules), resulting in a total of 350 Petri dishes, 1750 seeds. The nylon mesh was previously soaked and rinsed three times in distilled water. The mesh was placed inside Petri dishes (10 cm in diameter) containing a sufficient PEG solution to imbibe the seeds without fully covering them. The Petri dishes were incubated at a constant temperature of 25°C with a 12 h day^−1^ photoperiod. The Petri dishes were sealed with parafilm and placed inside plastic bags to avoid water loss. Radicular protrusion (germination) and solution levels were measured daily.

In Sections 2.4 and 2.5, final percentages of germination were arcsine transformed and analyzed using ANOVA and Tukey post hoc tests. The relations between seed mass, maximum germination, or total fatty acids in each elevational population with elevation, mean air temperature, or mean soil temperature were fitted to linear functions for regression analysis using Table Curve 2D, ver 5.0 (2000AISN Software Inc., Chicago, IL, USA). Principal component analysis (PCA) was performed to determine the associations between the concentration of each of the identified fatty acids and total fatty acids with seed mass, maximum germination, lag time, elevational population, and air and soil temperature of the different collection locations. Analysis was performed with Statgraphics, ver 5.0 (Statistical Graphics Corporation, Englewood Cliffs, New Jersey, USA).

### 2.6 Temperature Gradient Treatments

Germination treatments were carried out in a multimodal thermal incubator consisting of 15 solid aluminum cylinders (10.24 × 26.24 cm, diameter and length, respectively), each set at a different constant temperature. Each cylinder had five horizontal perforation holes (2.56 × 23.04 cm, diameter and length, respectively) to introduce 2.5 cm diameter test tubes, which were illuminated (12 h day^−1^) by two 40 W cool white fluorescent lamps (Sylvania). Each cylinder was maintained at one of 15 temperatures (5°C, 7°C, 9°C, 11°C, 14°C, 17°C, 21°C, 23°C, 25°C, 28°C, 31°C, 33°C, 35°C, 37°C, and 41°C). Fifty seeds were placed on removable agar plates (1% in distilled water) that covered half of the horizontal test tubes' capacity. For each elevational population, 75 tubes were sealed with polyethylene film to reduce water loss, and five replicates were placed per temperature treatment. In each cylinder, test tubes were rotated daily among the holes at each temperature. A total of 18,750 seeds were used. A full description of the multimodal thermal incubator is available in Márquez‐Flores et al. (1994). Radicle protrusion (germination) was recorded daily for 1 month. From this experiment, we obtained the percentages of germination in elevation and temperature and their lag times (times to initiate germination).

### 2.7 Thermal Time Model

The percentages of germination for each elevation and temperature were scaled to obtain at least 6–8 percentile subpopulations to calculate the thermal time (*T*_t_), the base temperature (*T*_b_; minimum temperature for germination), and the ceiling temperature (*T*_c_, maximum temperature for germination). The maximum percentage of germination observed in any of the treatments or replicates was considered 100% germination. Cumulative germination and time were fitted to exponential sigmoid curves (Orozco‐Segovia et al. 1996), and the times to reach each percentile subpopulation of 10%, 20%, 30%, 40%, 50%, 60%, 70%, and 80% at each temperature and elevation were determined from the function.

$$y=\frac{a}{1+{\mathrm{be}}^{-\mathrm{cx}}}$$

The germination rate (GR) was conventionally defined as the reciprocal of the time required to reach each of these percentages (Garcia‐Huidobro et al. 1982). GRs of each subpopulation along the temperature gradient were fitted to peak curves of the form:

$$y={\mathrm{ae}}^{-b\left(\frac{x}{c}-1\right)2}+\frac{1}{x-d}$$

where *a* is the maximal germination rate in each curve, *b* a parameter of the nonlinear Asymmetric Peak Function $\frac{1}{\sigma 2}$, *c* the temperature at which the GR is highest, and *d* is the intersection between the abscissa axis and temperature in the descending extreme of the Peak Function. The base (*T*_b_) and *T*_c_ temperatures were defined as the temperatures at the intersection between the tangent at the inflection points (at the first maximum and minimum derivatives of the Peak Function) and the abscissa axis. The *T*_t_ was defined as the reciprocal of the slope of the tangent at the inflection points of the Peak Function (Orozco‐Segovia et al. 1996).

The TableCurve software calculates the model, parameters, goodness of fit, the first maximum and minimum derivatives (1st_max_ and 1st_min_ derivatives), and their x‐values, where they were found (y‐values and ordinate to the origin of the tangent curve were calculated).

*T*_b_ and *T*_c_ were calculated as follows:

$$y_{\min}=a\times \exp\left(-b\times \left(\frac{x_{\min}}{c}-1\right)2\right)+\left(\frac{1}{x_{\min}-d}\right)$$

$$y_{\max}=a\times \exp\left(-b\times \left(\frac{x_{\max}}{c}-1\right)2\right)+\left(\frac{1}{x_{\max}-d}\right)$$

$${\text{ORD}}_{\min}=y_{\min}-\left(1\mathrm{st}\;{\text{derivative}}_{\min}\times x_{\min}\right)$$

$${\text{ORD}}_{\max}=y_{\max}-\left(1\mathrm{st}\;{\text{derivative}}_{\max}\times x_{\max}\right)$$

$$Tc=\frac{-{\text{ORD}}_{\min}}{1\mathrm{st}\;{\text{derivative}}_{\min}}$$

$$Tb=\frac{-{\text{ORD}}_{\max}}{1\mathrm{st}\;{\text{derivative}}_{\max}}$$

The percentile subpopulations and *T*_t_ for each elevation were fitted to linear models. Finally, *T*_t_, *T*_b_, and *T*_c_ were compared using ANOVA and Tukey tests. Statistical analyses and curve fittings were performed with Statgraphics, ver 5.0 (Statistical Graphics Corporation, Englewood Cliffs, NJ, USA), and all fittings were performed using TableCurve 2D. PCA was performed to determine associations between the lag time, maximum germination, seed mass, total lipid content, *T*_b_, *T*_c_, *T*_tsub_, *T*_tsup_, elevational population, and air and soil temperatures of the different collection sites. Analysis was performed with Statgraphics, ver 5.0 (Statistical Graphics Corporation, Englewood Cliffs, NJ, USA).

### 2.8 Cumulative Soil Temperature at 2345 m Above Sea Level

Further, soil temperature data were measured with a Hobo for each of the five microsites, which were buried under 3 cm of REPSA soil (2345 m a.s.l.). The temperature was recorded every 30 min for a week in the seed collection year, when the rainy season began in the same year as the *W. urens* seed collection. Five measurement days were chosen randomly, and temperatures from 8 to 18 h were considered to calculate the cumulative temperature from *T*_b_ to optimal temperature (T_o_), expressed as °C h and transformed to °C day. Subsequently, we estimated the number of days required to reach *T*_t_ for the 50% percentile subpopulation (*T*_t50%_).

## 3 Results

### 3.1 Relations Between Seed Traits, Elevation, Soil, or Air Temperatures

#### 3.1.1 Maximum, Mean and Minimum Soil Temperatures

The minimum, maximum, and mean air temperatures decreased as the elevation increased at rates of 0.67°C, 0.97°C, and 0.78°C for each 100 m elevation, respectively (SMN 2023a, 2023b); while the soil temperatures changed at rates of 0.71°C, 0.79°C, and 0.76°C for each 100 m (minimum, maximum, and mean temperatures).

**FIGURE 1:**
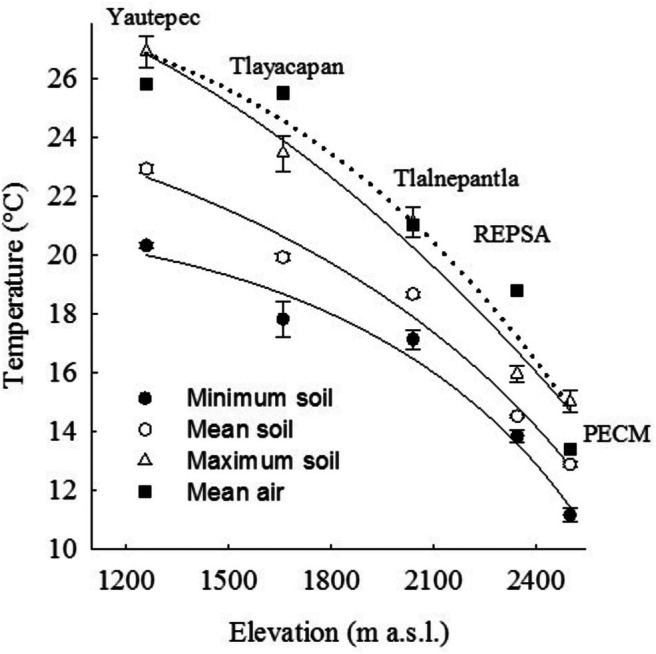
Minimum, mean, and maximum soil temperatures and mean air temperature at elevations where each elevational population of *Wigandia urens* seeds were collected in Central Mexico, close to the localities indicated in this figure. The three lowest sites are in Morelos state, and the two highest are in Mexico City. Mean ± SD.

At all elevations, the mean air temperature was comparable to the maximum soil temperature (Figure 1).

#### 3.1.2 Seed Traits

The seeds of *Wigandia urens* were 0.476–0.532 mm long and 0.343–0.381 mm wide; no significant differences between populations (*p* > 0.05). The mean number of seeds per capsule was 1160 ± 361. The number of capsules per inflorescence was 26 ± 6.3, and the number of inflorescences per panicle was 10.73 ± 1.8. The estimated mean number of seeds per panicle was 328,941.

**FIGURE 2:**
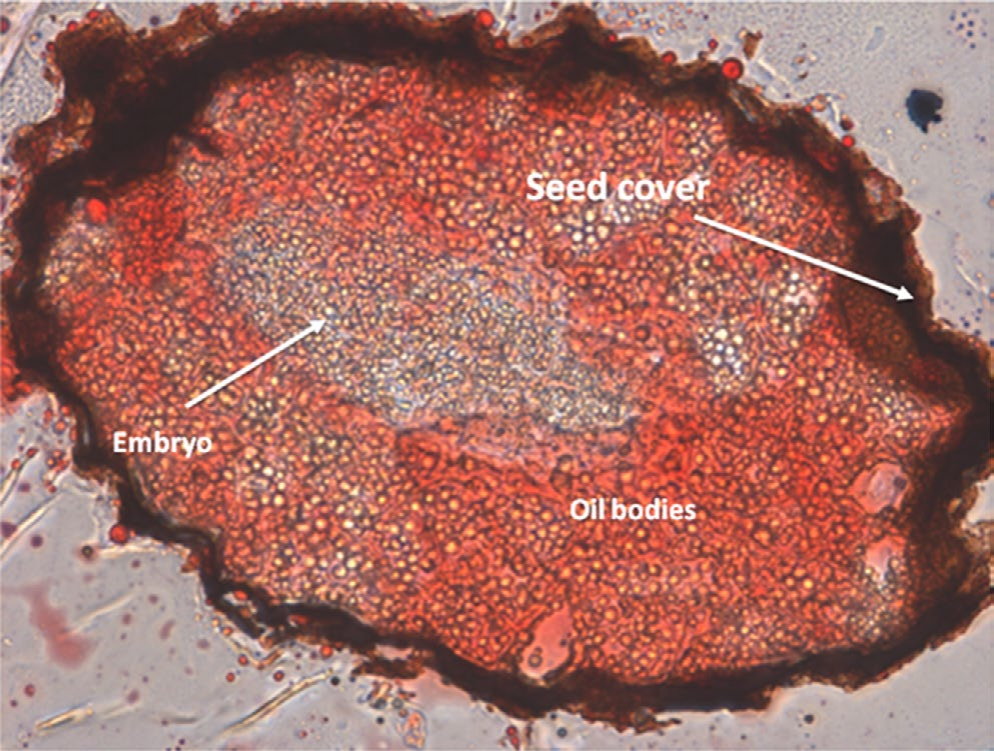
Transverse section of a *Wigandia urens* seed, stained with Oil Red. Multiple oil bodies are observed.

The seed mass was 0.019–0.027 mg and the seed moisture content on a wet basis was 16.84% ± 13.01%. The WC calculated as an oil‐free percentage (of) on a fresh basis was 24.34%. The presence of oil bodies inside the seeds is shown in Figure 2.

In all elevations, total fatty acid content was highest at 2500 m a.s.l. (55.747 mg g^−1^). Primarily determined for the concentrations of the unsaturated fatty acids, Linoleic (67.969–272.063 mg g^−1^) and Oleic (84.317–215.242 mg g^−1^). In the seeds from the other elevations, this value ranged from 30.816 to 19.19 mg g^−1^. The saturated fatty acids, Palmitic and Stearic, ranged from 14.800 to 47.185 and 8.227 to 19.193 mg g^−1^, respectively. In all seed populations, Nonadecilic acid presented the lowest content (3.690–4.76 mg g^−1^) (Table 2).

**TABLE 2:** Fatty acids content found in the *Wigandia urens* 's seeds from different elevational populations of Central Mexico.

Elevational population m a.s.l.	Palmitic (mg g^−1^)	Linoleic (mg g^−1^)	Oleic (mg g^−1^)	Stearic (mg g^−1^)	Nonadecilic (mg g^−1^)	Total (%)
1260	24.232	90.525	146.011	10.988	3.702	27.546
1660	14.800	67.969	97.199	8.227	3.709	19.190
2040	23.611	106.627	127.372	11.825	3.824	27.326
2345	30.506	174.380	84.317	14.193	4.760	30.816
2500	47.185	272.063	215.242	19.290	3.690	55.747

**FIGURE 3:**
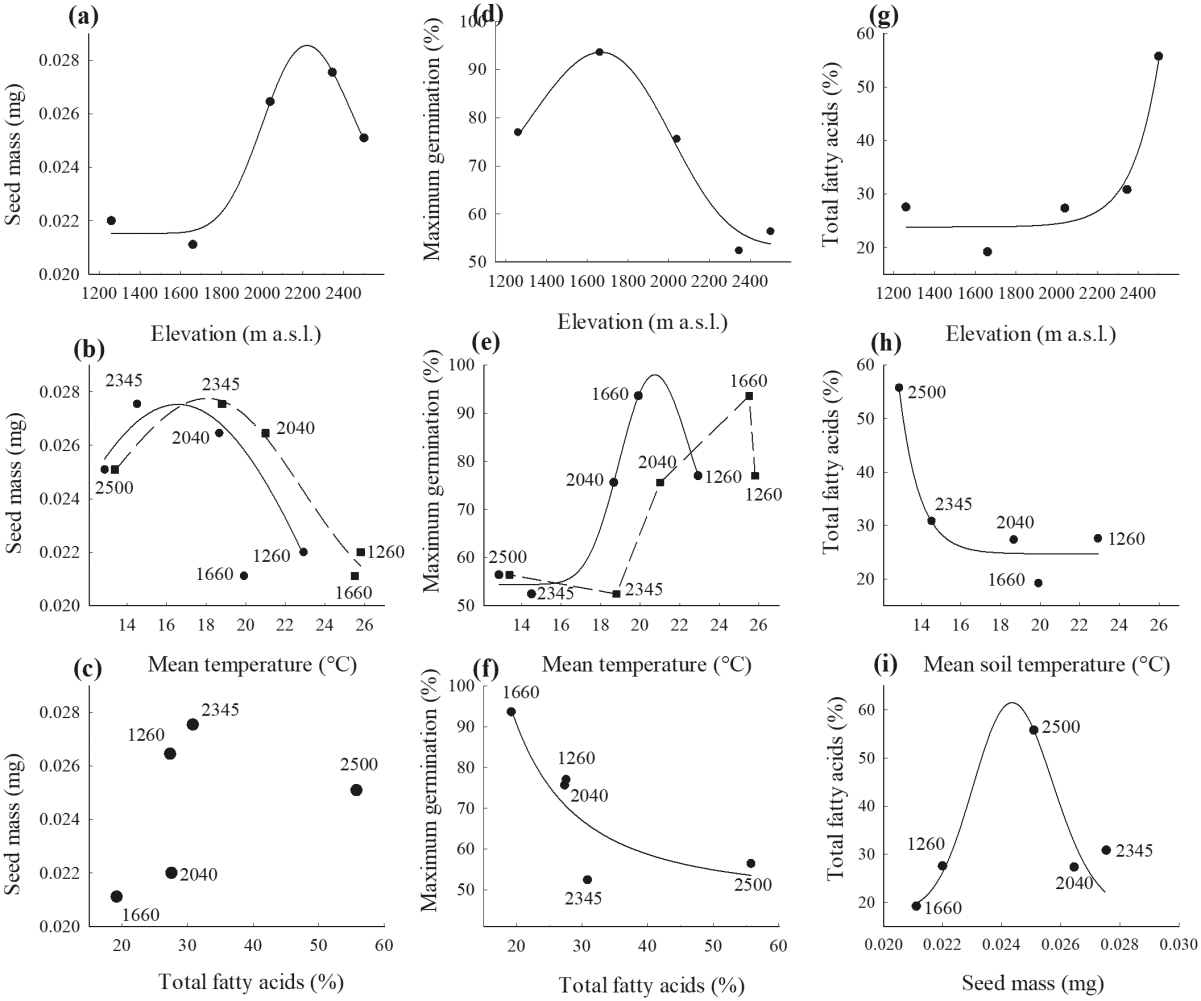
Relations between seed mass of *Wigandia urens* versus altitude (a), mean air temperature (b), and total fatty acids content (c); maximum germination percentage versus elevation (d), mean soil temperature (e), and total fatty acids content (f); and total fatty acids content versus elevation (g), mean soil temperature (h) from their collection sites or seed area (i). The numbers within the panels indicate the elevational seed population.

Relations between seed traits and some climate elements were fitted to the models shown in Figure 3 and Appendix 1. The highest seed mass along the elevational gradient was skewed toward the three higher elevations (Figure 3a). Seed mass was also skewed to the lowest air and soil temperatures (Figure 3b). Maximum germination was skewed toward lower elevations (Figure 3d) and the highest soil temperatures (Figure 3e). This trend was also observed with air temperature, but the data did not fit any function. Maximum germination decreased as total fatty acid content increased (Figure 3f).

Despite the high presence of oil bodies inside the seed (Figure 2), total fatty acid concentrations did not explain seed mass (Figure 3c). The total fatty acid content was highest at 2500 m (Figure 3g), resulting in the lowest value at the lowest mean soil temperature (Figure 3h). A log‐normal model described the relation between total fatty acids and seed mass, with the highest value in seeds from 2500 m (Figure 3i). Outliers were excluded from the models in all cases but shown in the figures.

### 3.2 Germination Capacity

For each elevation, seed age and gibberellic acid treatments had no significant effect (*p* > 0.5) on seed germination. We did not identify the presence of seed dormancy; mean germination in each elevational seed population was maintained as follows: 1260 m = 77.58% ± 5.96%, 1660 m = 89.75% ± 5.95%, 2040 m = 66.83% ± 12.53%, 2345 m = 54.5% ± 13.24%, and 2500 m = 57.58% ± 13.01%. There were no significant differences (*p* > 0.5) between GA_3_ treatments or seed age. Therefore, we used two‐year‐old seeds for all subsequent treatments.

**FIGURE 4:**
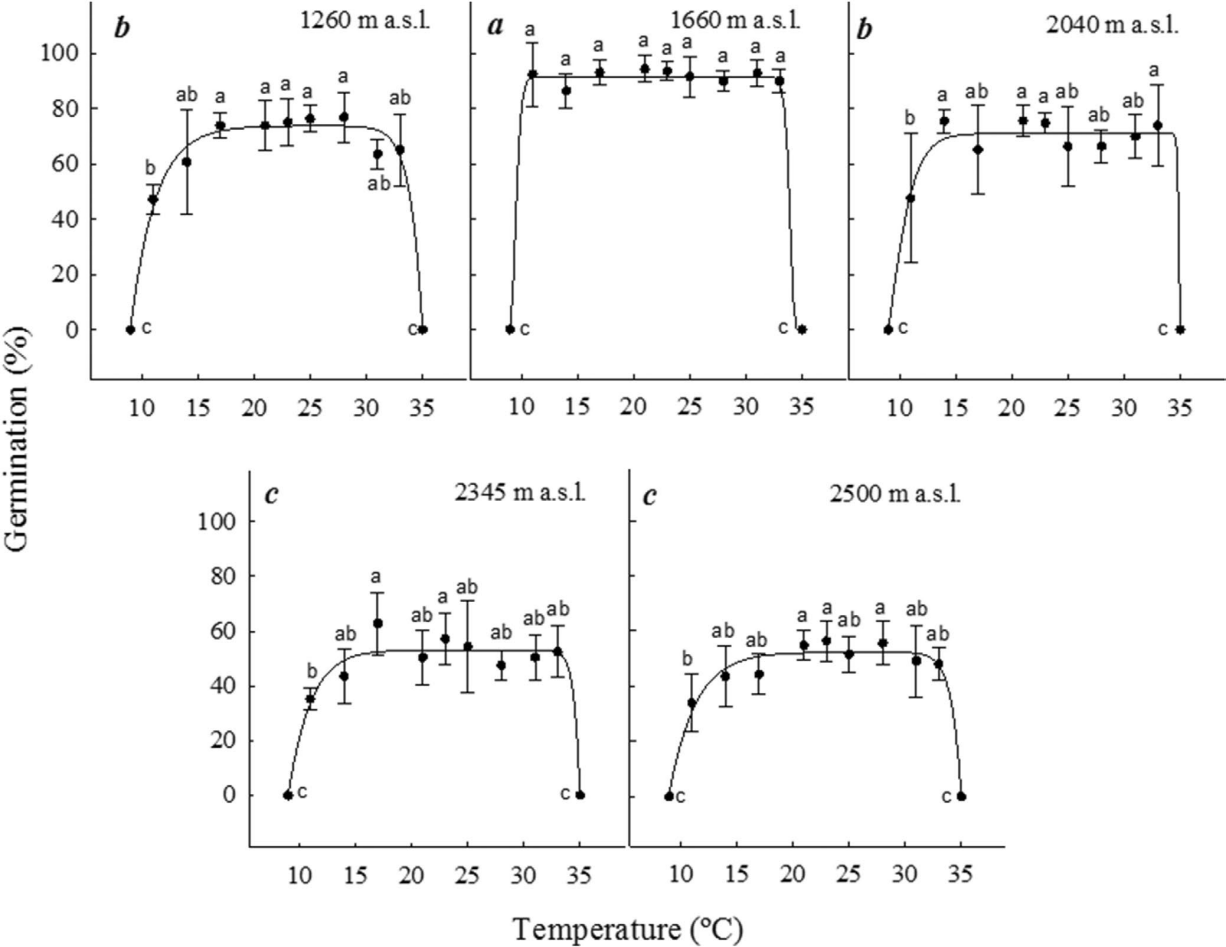
Maximum germination percentage of *Wigandia urens* seeds collected from different elevational populations in Central Mexico and germinated on a temperature gradient. The letters indicate significant differences between temperatures, and italic letters indicate significant differences between elevational populations. Mean ± SD.

Germination in the gradient was significantly affected by germination temperature (*F* = 369.67; df = 10, 268; *p* = 0.00001), elevation (*F* = 158.83; df = 4, 268; *p* = 0.00001), and their interaction (*F* = 5.08; df = 40, 268; *p* = 0.00001). Germination was null at 10°C and 35°C and occurred from 14°C to 33°C without significant differences within each elevational population. Nevertheless, between elevations, mean maximum germination was significantly lower in the seed populations of 2345 and 2500 m (52.3% ± 5.8% and 50.4% ± 4.9%, respectively) than at 1660 m (91.5% ± 2.6%). Significantly low germination percentages were found at 11°C in all seed populations (mean value = 41% ± 7.4%) except in the 1660 m seed population, which maintained high germination at all temperatures (Figure 4, Appendix 2). All non‐germinated seeds were neither rotten nor showed evidence of death.

**FIGURE 5:**
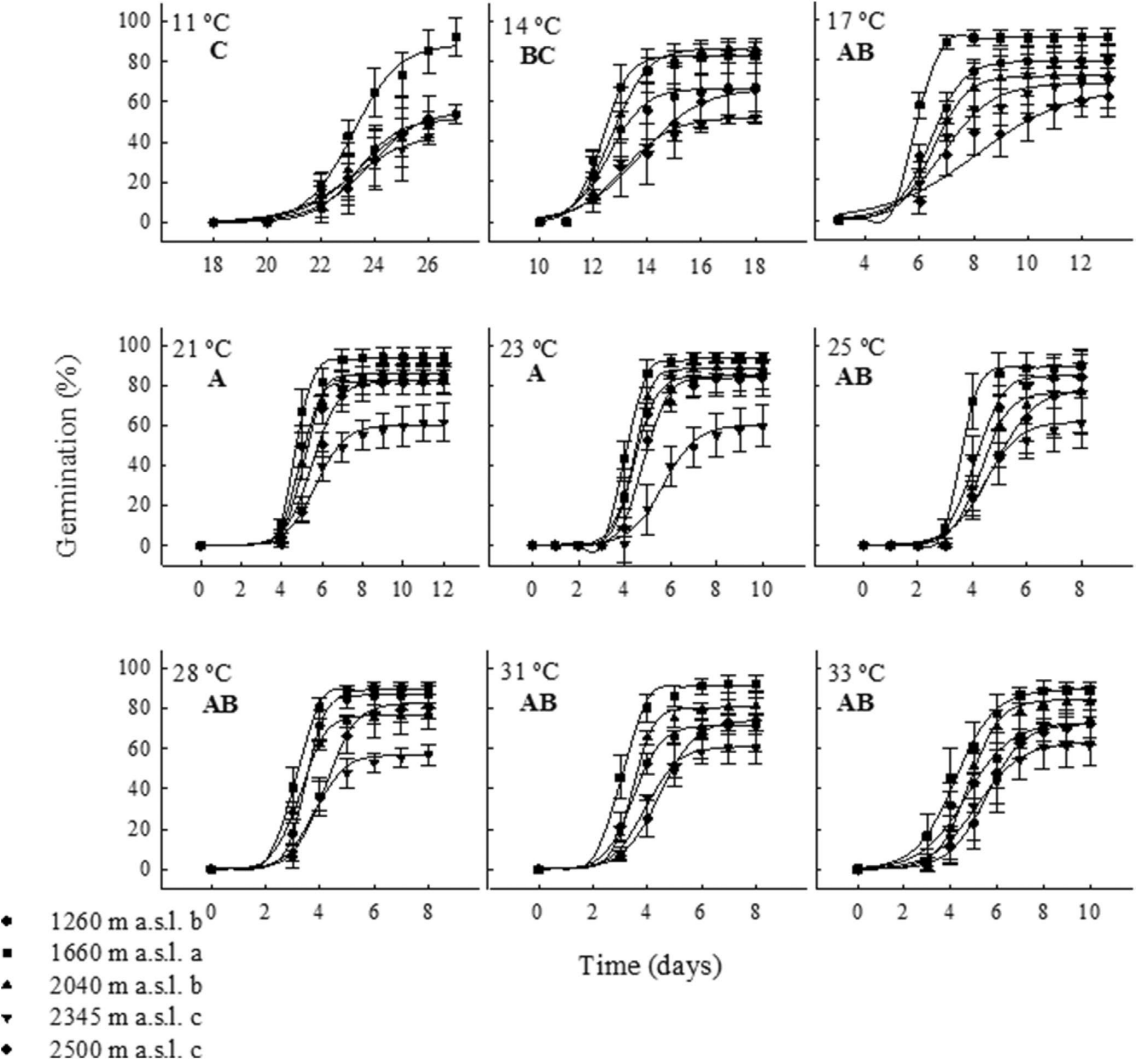
Cumulative germination percentage of *Wigandia urens* seeds collected from different elevational populations in Central Mexico and germinated in a temperature gradient. Bold capital letters indicate significant differences between temperatures, and small letters indicate significant differences between elevational populations. Mean ± SD.

Elevation, germination temperature, and their interaction had a significant effect on lag time (Figure 5, Appendix 2). The significantly lowest elevational mean value was found in the 1660 population (6.5 ± 5.86 days), and the highest (7.17 ± 6.05 days) in the 2500 population. Generally, higher germination temperatures reduced lag time; thus, germination at 31°C or 28°C reduced lag time to 3 ± 0 and 3.08 ± 0.27 days, respectively, compared to those observed at 11°C or 14°C (21.72 ± 0.93 and 12.08 ± 0.4 days, respectively).

In *W. urens* seeds, we found the presence of three saturated fatty acids (palmitic, stearic, and nonadecilic) and two unsaturated fatty acids (oleic and linoleic), whose concentrations varied between elevational populations. The total lipid content ranged from 19.9% to 55.7%, showing an increasing trend as elevation increased, except for 1660 m, which had the lowest percentage of lipids.

**FIGURE 6:**
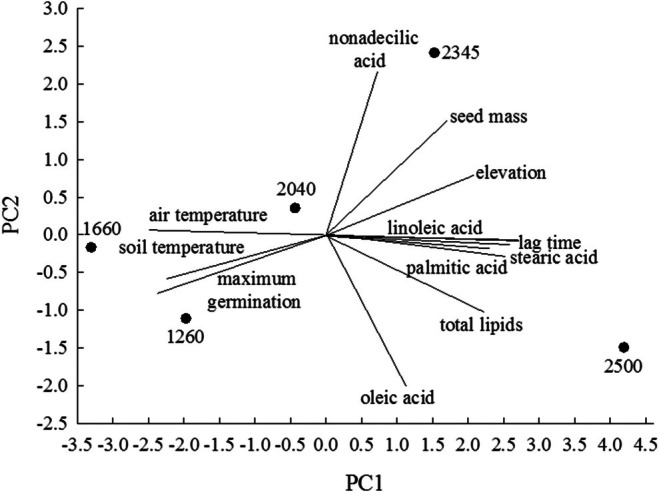
Biplot for PCA to determine the associations between fatty acids contained in the seeds of *Wigandia urens*, total fatty acids, theseed mass, maximum germination, thelag time, the elevation of the seed populations, and soil temperatures at each seed collection. The lines show the negative or positive associations between the different variables.

Principal components analysis showed that the variability contained in the two main axes, 72.70% (PC1) and 19.61% (PC2), explained 92.32% of the cumulative variance (Figure 6). According to PCA, the concentrations of each fatty acid (linoleic, nonadecilic, oleic, palmitic, and stearic), the total percentage of fatty acids, seed mass, elevational population, and lag time were placed on the positive side of PC1, suggesting a positive association between these attributes of the seeds, while maximum germination and soil and air temperatures from collection locations were on the negative side of PC1, suggesting a negative association between seed traits and those environmental factors. Oleic acid was placed on the negative side of PC2, suggesting a negative association with the other variables.

### 3.3 Germination Percentage in the Osmotic Potential Gradient

**FIGURE 7:**
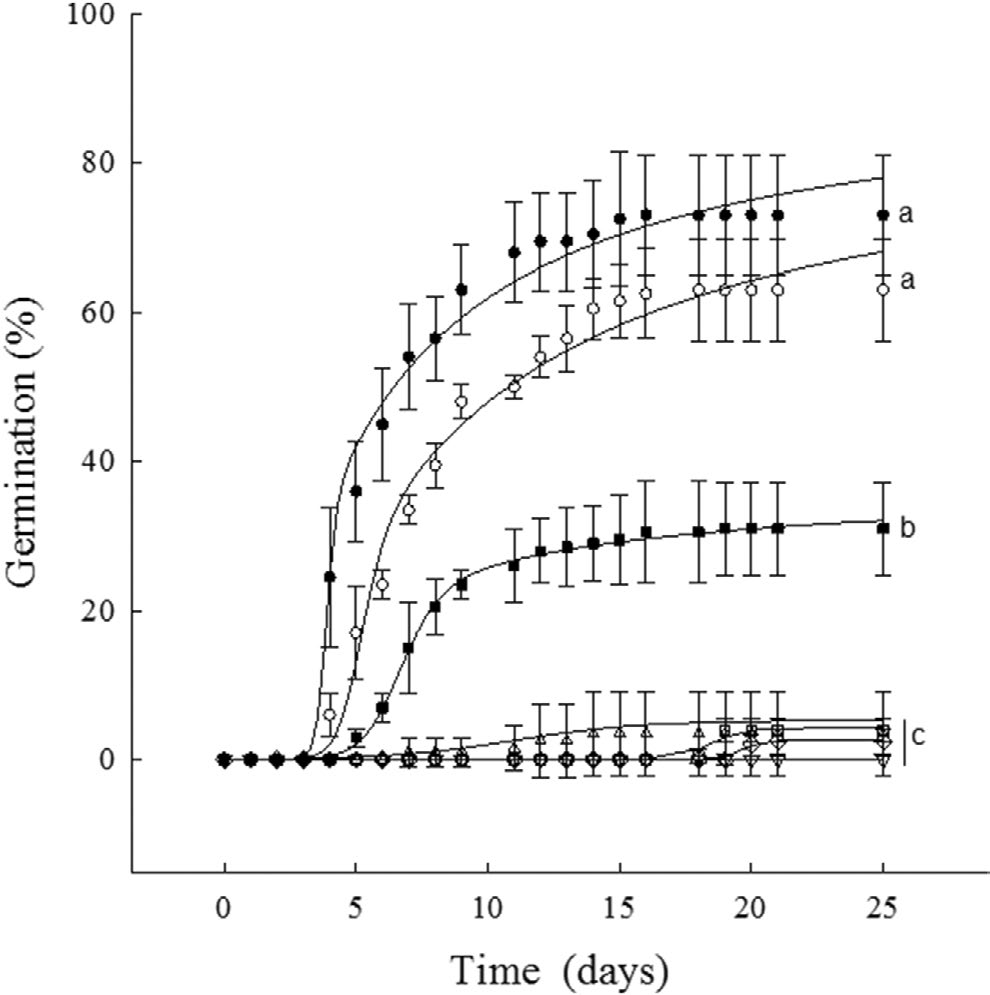
Germination percentage of *Wigandia urens* seeds in an osmotic potential gradient: (●) 0 MPa, (○) –0.5 MPa, (■) –1 MPa, (△) –1.5 MPa, (□) –2 MPa, (◊) –2.5 MPa, (▽) –3 MPa. Mean ± SD.

The osmotic potential significantly affected seed germination (*F* = 85.92; df = 6, 27; *p* < 0.0001). The seeds required osmotic potentials from 0 to 0.5 MPa to achieve 73% ± 8.1% to 63% ± 6.8%. Significantly lower germination was obtained at osmotic potentials of −1.0, −1.5, −2.5, and −3 MPa (31% ± 6.2%, 4% ± 1.6%, 2.5% ± 1.7%, and 0%, respectively; Figure 7).

### 3.4 Thermal Time Model

**FIGURE 8:**
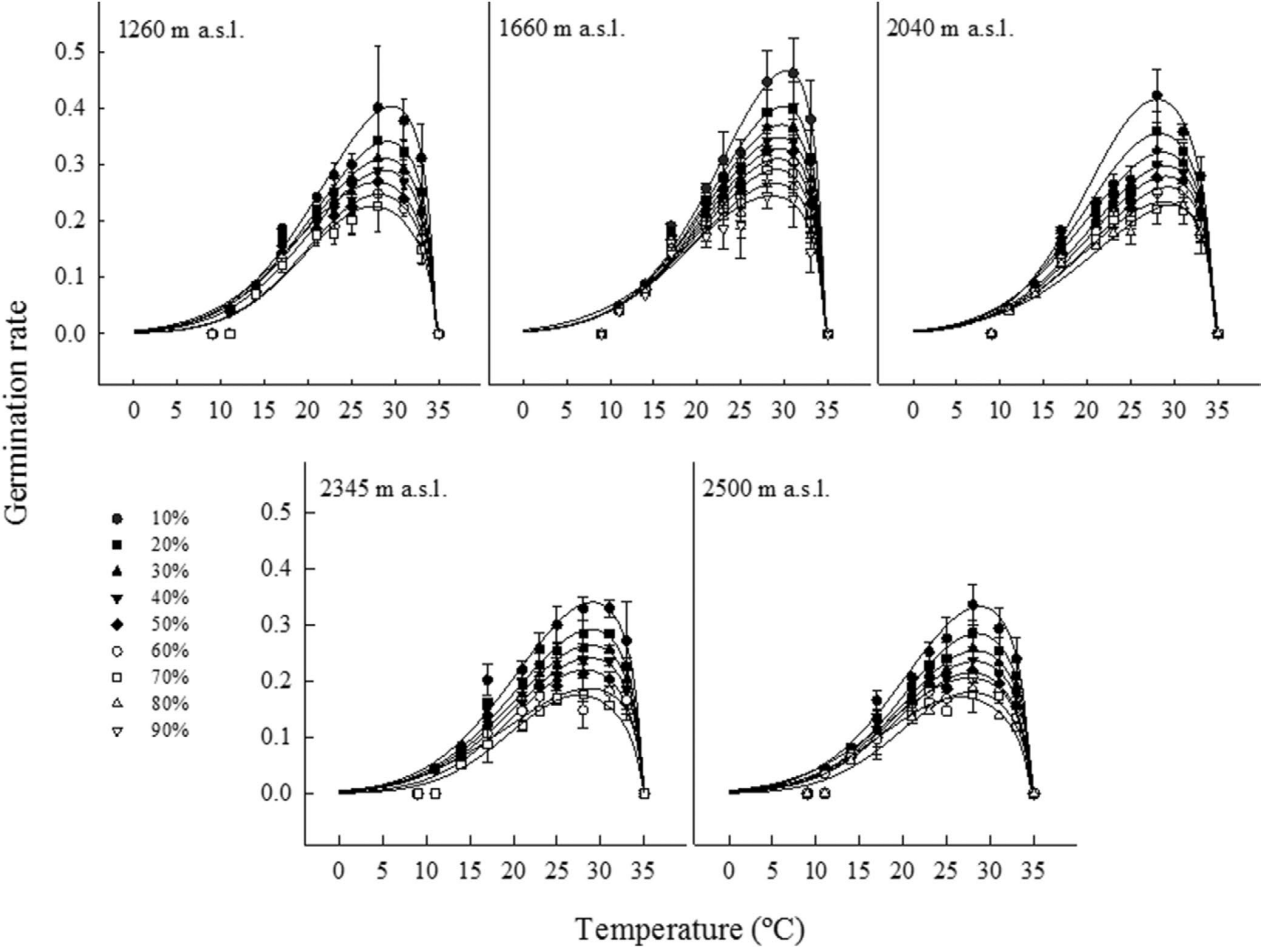
Germination rates for the percentile subpopulations of *Wigandia urens* seeds collected from different elevational populations in Central Mexico and germinated in a temperature gradient. Mean ± SD.

Elevation and the percentile subpopulation significantly affected *T*_b_, *T*_c_, and *T*_t_ at temperatures lower than optimal (thermal time suboptimal; *T*_tsub_) and higher than optimal (thermal time supraoptimal; *T*_tsup_). In no case was the interaction between these factors significant (Figure 8, Appendix 3).

#### 3.4.1 Optimal Temperatures

Maximum germination percentages and rates were found in the interval 28°C–31°C for all elevational populations (Figure 4).

#### 3.4.2 Base Temperatures

Among elevational populations, *T*_b_ for germination of *W. urens* seeds ranged from 9.81°C to 12.48°C. In seeds collected at 2345 m (10.24°C) and 1660 m (11.16°C), *T*_b_ values were significantly the lowest and highest, respectively (Table 3).

**TABLE 3:** Temperature requirements of the seeds of *Wigandia urens* , from different elevational populations of Central Mexico, to achieve different germination percentile populations.

Elevational population (m a.s.l.)	Percentile subpopulation (%)	Base temperature (°C)	Ceiling temperature (°C)
1260	10	10.98 ± 0.54 a	34.89 ± 0.13 ns
	20	10.59 ± 0.58 ab	34.89 ± 0.13 ns
	30	10.40 ± 0.55 ab	34.88 ± 0.12 ns
	40	10.36 ± 0.14 ab	34.85 ± 0.11 ns
	50	10.18 ± 0.55 b	34.83 ± 0.12 ns
	60	9.81 ± 0.62 b	34.82 ± 0.11 ns
	70	10.41 ± 1.44 ab	34.85 ± 0.14 ns
1660	10	12.48 ± 0.46 a	34.83 ± 0.12 ns
	20	11.26 ± 0.74 b	34.80 ± 0.11 ns
	30	10.91 ± 0.52 bc	34.79 ± 0.11 ns
	40	10.94 ± 0.45 bc	34.79 ± 0.11 ns
	50	10.68 ± 0.36 bcd	34.79 ± 0.11 ns
	60	10.68 ± 0.18 bcd	34.79 ± 0.11 ns
	70	10.37 ± 0.38 cd	34.79 ± 0.10 ns
	80	10.30 ± 0.47 d	34.80 ± 0.10 ns
2040	10	11.53 ± 0.54 a	34.78 ± 0.05 ns
	20	11.08 ± 0.40 ab	34.81 ± 0.08 ns
	30	10.87 ± 0.26 ab	34.82 ± 0.08 ns
	40	10.74 ± 0.53 ab	34.83 ± 0.10 ns
	50	10.53 ± 0.75 b	34.80 ± 0.10 ns
	60	10.46 ± 0.61 b	34.85 ± 0.11 ns
	70	10.67 ± 0.84 ab	34.74 ± 0.01 ns
2345	10	10.39 ± 1.09 ns	34.89 ± 0.13 ns
	20	10.26 ± 0.50 ns	34.89 ± 0.13 ns
	30	10.10 ± 0.40 ns	34.89 ± 0.13 ns
	40	9.99 ± 0.57 ns	34.89 ± 0.13 ns
	50	10.71 ± 0.77 ns	34.93 ± 0.11 ns
	60	10.02 ± 1.55 ns	34.88 ± 0.15 ns
2500	10	11.12 ± 0.51ab	34.89 ± 0.13 ns
	20	10.84 ± 0.53 ab	34.93 ± 0.10 ns
	30	11.02 ± 0.51 ab	34.98 ± 0.02 ns
	40	10.89 ± 1.15 ab	34.97 ± 0.02 ns
	50	10.82 ± 0.94 ab	34.97 ± 0.04 ns
	60	10.45 ± 1.05 b	34.79 ± 0.10 ns
	70	12.00 ± 0.49 a	34.74 ± 0.07 ns

*Note:* Letters indicate significant differences within each elevational population. Mean values ± SD, *n* = 5, ns, non significant.

#### 3.4.3 Ceiling Temperatures

The significantly lowest value was found in seeds from 1660 m (34.80°C) and the significantly highest value in seeds from 2345 m and 2500 m (34.90°C and 34.92°C, respectively; Table 3).

#### 3.4.4 Suboptimal Thermal Time

**FIGURE 9:**
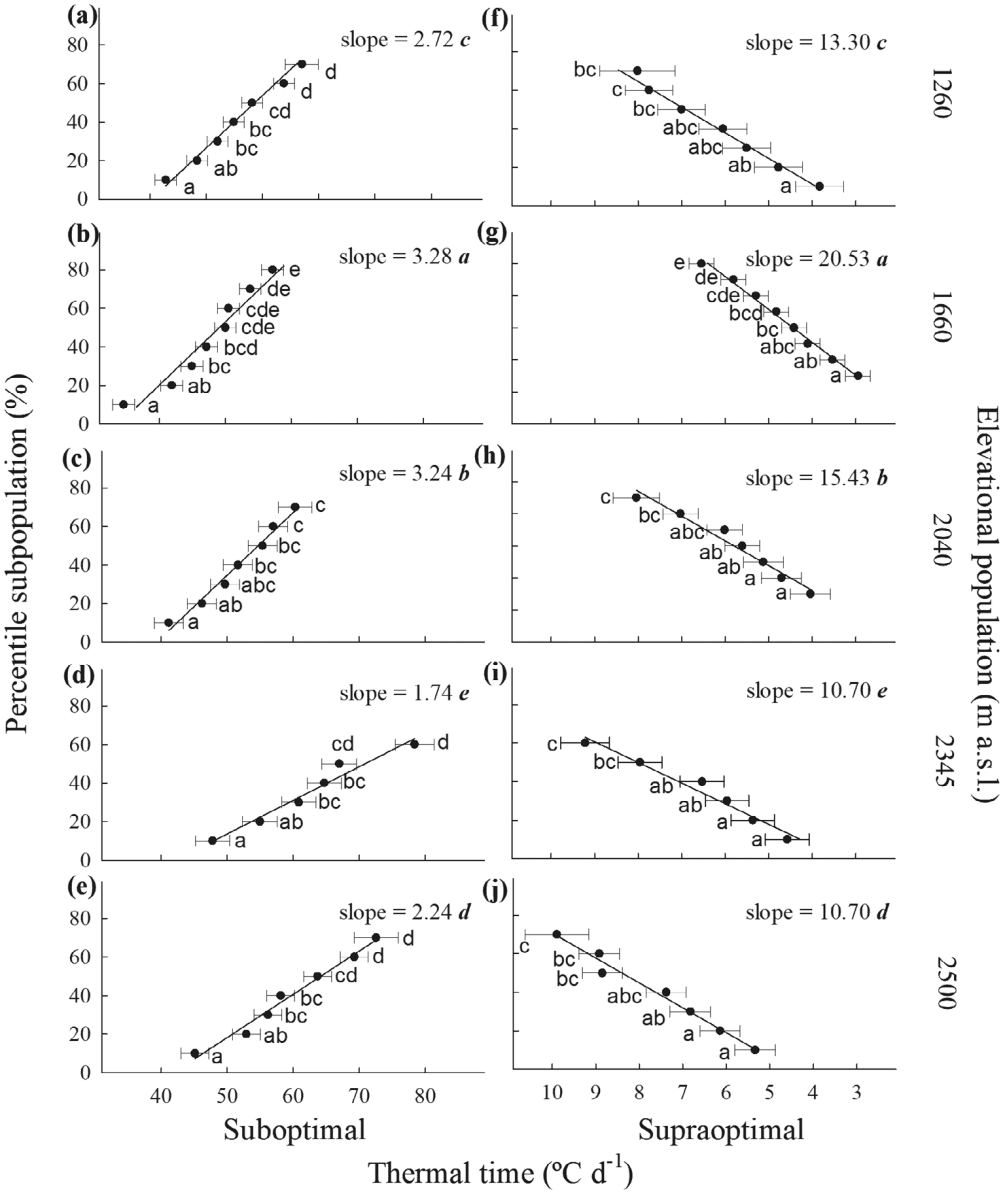
Thermal times at suboptimal (a–e) and supraoptimal (f–j) temperatures for the percentile germination subpopulations of seeds of *Wigandia urens* collected in different elevational populations in Central Mexico. The letters indicate differences within elevational populations, mean ± SD Italic bold letters indicate differences between elevational populations for slopes from the relationships between percentile subpopulations and thermal times fitted to models.

The *T*_tsub_, values were between 44.85°C day (1660 m) and 62.27°C day (2345 m), with seeds from the other elevational populations showing significant intermediate values without significant differences between them (Figure 9a–e, Appendix 3). The slopes of the linear regressions fitted to *T*_tsub_ varied from 3.28% (°C day)^−1^ to 1.74% (°C day)^−1^ (at 1660 and 2345 m, respectively; *F* = 709.82; df = 4,14; *p* = 0.00001; Figure 9a–e).

#### 3.4.5 Supraoptimal Thermal Time

For *T*_tsup_, the significantly lower value was 4.18 (at 1660 m), while the highest values were 6.60°C and 7.24°C day (at 2345 m and 2500 m, respectively), without differences between them. For seeds from the other elevations, *T*_tsup_ had intermediate values, without significant differences between them (Figure 9f–j, Appendix 3). Slopes from the percentile subpopulations related to *T*_tsup_ showed a decreasing trend from 1660 to 2345 (20.53%, 15.43%, and 10.70% (°C day)^−1^, respectively). The values were significantly different between all elevations (*F* = 709.82; df = 4,14; *p* = 0.00001; Figure 9f–j).

**FIGURE 10:**
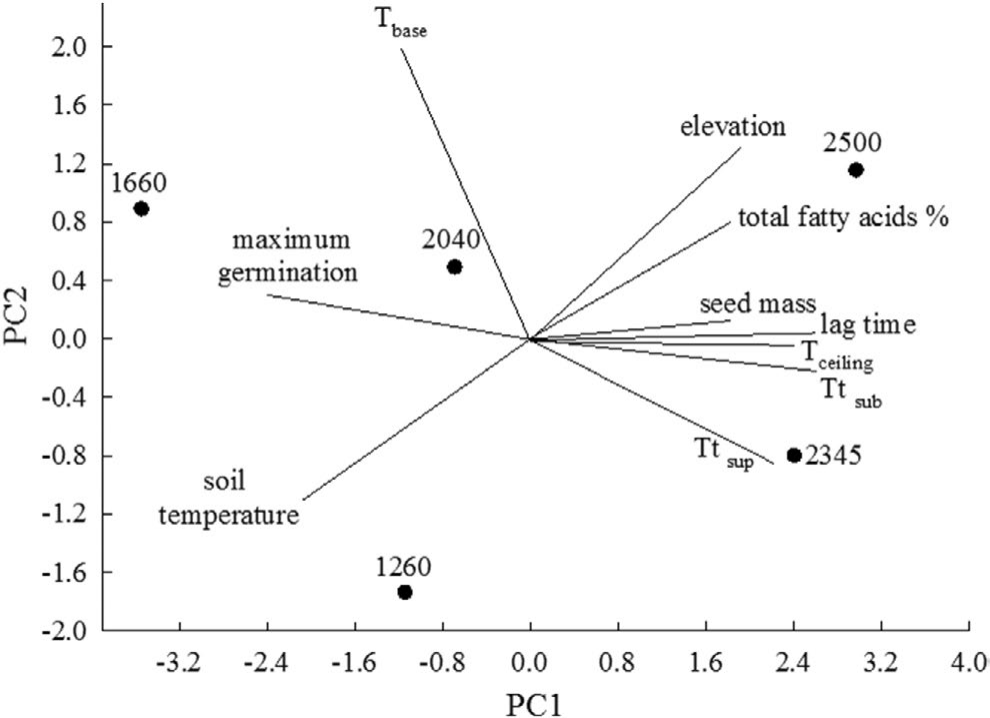
Biplot for PCA to determine in *Wigandia urens* seeds the associations between seed mass, percentages of total fatty acids, maximum germination, lag time, base and ceiling temperatures, suboptimal and supraoptimal thermal times (*T*_tsub_ and *T*_tsup_, respectively), soil temperature, and elevation in each seed collection location. The lines show the negative or positive associations between the different variables.

In the PCA, the explained variability of each of the two main axes was 72.38% (PC1) and 15.07% (PC2), which explained 87.45% of the cumulative variance (Figure 10). According to the PCA, two data groups were observed, determined by their position on PC1. On the positive side of this PC, there was a positive association between elevation, total lipid percentage, *T*_tsup_, *T*_tsub_, *T*_c_, seed mass, and lag time. All these variables were negatively associated with soil temperature, *T*_b_, and maximum germination, which were located on the negative side of PC1.

### 3.5 Cumulative Soil Temperature

At the beginning of June, when the rainy season starts, the cumulative temperature over 24 h was 284.1 ± 17.71°C day^−1^. Considering the temperatures between *T*_b_ and *T*_o_, the cumulative temperature during a diurnal period was 25.6 ± 7.95°C day^−1^. Thus, to reach T_t50_ = 62.5°C day^−1^, approximately 2.42 days might be necessary.

## 4 Discussion

### 4.1 Relationships Between Seed Traits, Elevation, Soil, and Air Temperatures

The five *Wigandia urens* seed populations collected along the elevational gradient (1260–2500 m a.s.l.) exhibited high germination capacity (56%–94%, Figure 4) in a wide thermal window (11°C–33°C) and high optimal temperatures (28°C–31°C). This trait indicates that *W. urens* is a thermophilic species, which may enable it to withstand thermal stress in afforested areas and cope with the effect of CC during germination.

In this research, the maximum germination (94%–56%, Figure 4) depended on the elevational population of seeds and the temperature (11°C–33°C). The elevations with the lowest germination were 2500 m and 2345 m. Germination did not occur at the lowest or highest temperatures tested. Conversely, seeds of *W. urens* collected at 2345 m a.s.l. in 2013–2014 germinate at 10°C and 35°C in more than 50% (Martínez‐Villegas et al. 2018). These seeds were collected in a hotter year (mean temperature in May: 19.7°C) than in 1998 (14.5°C). Differences in thermal window and maximum seed germination for each temperature at the same elevation (≈2345 m) suggest the presence of maternal effects, such as the acquisition of temperature stress memory during seed development (Donohue 2009; Peng et al. 2019). However, the limits of the wideness of the thermal window for germination of the species are determined by their phylogenetic history (Donohue et al. 2010). The prospects for using *W. urens* in restoration or assisted migration projects are promising because the average temperatures within the studied elevational interval differed by 10.7°C ± 1.37°C between the extreme elevations, a value higher than most of the highest estimations (6°C) predicted by the CC models for the next century (Bendou et al. 2022). Consequently, this species, also known as a bioengineer (Lira‐Caballero et al. 2018), might be capable of mitigating the effects of CC (Vacek et al. 2023) and improving biodiversity by modifying soil temperature, light, nutrients, and moisture through plant cover traits and litter production (Liu et al. 2024). For example, *W. urens* produces litter (75.5 ± 25 g m^−2^), and beneath its canopy, the mean photosynthetic photon flux density is reduced from approximately 1500 μmol m^−2^ s^−1^ (on a sunny day) to 135.2 ± 67.6 μmol m^−2^ s^−1^, depending on the distance from the *W. urens* tree. Further, under its projected shade, the mean temperature varies between 16.4 ± 7.3 and 19.9°C ± 9.5°C on a sunny day, and in an open site from 19.9°C ± 9.5°C to 20.6°C ± 9.9°C (unpublished data).

### 4.2 Germination Capacity

The most important environmental factor determining the germination capacity of *W. urens* was temperature (both soil and air). *W. urens* seeds germinated at high percentages of 73% at 0 MPa and 63% at −0.5 MPa. If the highest germination percentage is considered as 100%, then 5.84% of seeds germinated at −2 MPa. Though small, this percentage is significant, given the species' high seed production and the fact that seed loss and seedling mortality in the field are high due to multiple factors (De La Cruz et al. 2008). This species grows in stressful seasonal environments, such as lava fields, as in the REPSA (2345 m a.s.l.) and PECM (2500 m). In these areas, soil accumulation varies between sandy clay soil and sandy clay loam (García‐Aguirre et al. 2007), whose field capacities might be relatively high (−0.013 to−0.021 MPa, respectively; Phogat et al. 2024). Precipitation, water evaporation, and soil type primarily affect soil water availability, which is critical for seed germination. The germination of *W. urens* obtained at −1 and −1.5 MPa suggests a high tolerance to water stress. The seed's response to different osmotic potentials might lead to a staggered germination strategy capable of responding to the erratic distribution of rain occurring in the REPSA, at the beginning of the rainy season (Vivar‐Evans et al. 2006; Olvera‐Carrillo et al. 2009) or due to CC (Bendou et al. 2022).

The presence of dormancy has not been demonstrated in this species. However, in each population, a percentage of seeds did not germinate (Figure 4) even with gibberellins after 2 years of storage. At 25°C, ethylene at 0.01 M also does not induce germination in 20%–40% of *W. urens* seeds collected at 2292–2365 m a.s.l., in 2014 (Martínez‐Villegas et al. 2018). We also observed well‐developed seeds in 100% of X‐ray images, suggesting the presence of deep primary dormancy (*sensu* Baskin and Baskin 2014) in a fraction of the seed population. In this species, non‐germinated seeds can remain on a wet substrate for several months without evidence of decay (Alma Orozco‐Segovia, personal observations). Further, González‐Zertuche et al. (2001) and Gamboa‐deBuen et al. (2006) report that *W. urens* seeds can remain buried without germinating, but after unearthing, pre‐burial germination percentages increase, indicating that the non‐germinating fraction of seeds loses dormancy in the soil, as occurs with other species (Orozco‐Segovia et al. 2014). These observations emphasize the importance of both nondormant and deep dormant seed fractions in the *W. urens* seed bank (*sensu* Chambers and MacMahon 1994; Olvera‐Carrillo et al. 2009). Dormant seeds capable of remaining in the seed bank for extended periods may be essential to avoid the loss of seeds and seedlings due to unpredictable rain due to CC. We calculated that each panicle produces approximately 329,000 seeds, but seed loss and seedling mortality in the field are high due to multiple factors (De La Cruz et al. 2008). Thus, all seeds must face CC or unpredictable environments (Kildisheva et al. 2020).

Seed mass, elevation, fatty acid content, and lag time had a positive association between them and a negative association with both mean soil and air temperatures and with maximum germination (Figure 6). Moreover, the distribution of these traits was not linear; their highest or lowest values were found at the extremes of the elevation gradient (Figure 3), as observed in *Pinus brutia* (Tonguç et al. 2022). This distribution is related to the presence and/or concentrations of fatty acids and total reduced sugars at lower elevations or substances with protective functions at higher elevations. *W. urens* seeds were considered oil seeds due to their WC_of_ was > 17% (*sensu* Caddick 2002). They also exhibited abundant oil bodies, containing fatty acids common among seeds (Linoleic, Oleic, Palmitic, and Stearic); only the Nonadecilic acid was an unusual finding in seeds, as seen in *Spondias tuberosa* (Souza de Freitas et al. 2024). The saturated: unsaturated fatty acid ratio determines germination and plant growth; in *W. urens*, this ratio was the lowest (0.136) at 2500 m. Similar to seed mass, these traits exhibited an increasing trend from low to high elevations, as found in tropical species growing at high elevations (Herrera‐Legarreta and Almeida‐Leñero 1994; Tonguç et al. 2022) or where air temperatures are lower. However, in *W. urens*, seed mass decreased again at 2500 m, an elevation close to its distribution limit in Central Mexico (Rzedowski and Rzedowski 2019). Low saturated: unsaturated fatty acid ratios have been associated with temperate areas, while high ratios are more common in warm areas (Linder 2000; Sanyal and Decocq 2016). In *W. urens*, the unsaturated acids increased notably with elevation (from 246.56 mg g^−1^ at 1260 m a.s.l. to 487.87 mg g^−1^ at 2500 m), possibly due to the decrease in temperature with elevation (Harris and James 1969). However, saturated: unsaturated fatty acid ratios and concentrations did not exhibit a significant linear distribution with elevation, probably due to the microsite characteristics at each collection site, their temperatures primarily determining the fatty acid concentrations (Lajara et al. 1990; Linder 2000).

### 4.3 *T*_b_, *T*_c_, and Thermal Time

In *W. urens* , among percentile subpopulations, both within and between elevations, *T*_c_ and *T*_b_ showed few differences, with variations not exceeding 1°C–2°C. This suggests that the ability to germinate at high or relatively low temperatures is an inherent adaptation of the species, as explained by García‐Huidobro et al. (1982). Conversely, there was a wide temperature interval between *T*_b_ and *T*_c_ (≈25°C), with the seed germination rate declining sharply in the supraoptimal range (≈34°C–35°C). Despite this, at 33°C, the percentage of germination ranged from 40%–90%, depending on the elevational population. *T*_b_ and *T*_c_ confirmed that *W. urens* has tropical affiliation, although it grows in both tropical and temperate climates at high and low elevations (Pérez‐Estrada et al. 2000).

Thermal time in the suboptimal range was significantly higher at 2345 m than in the other populations; in contrast, in the supraoptimal range, there was a decreasing trend in elevation, except for *T*_t_ at 1260 m, which had a low value relative to higher elevations. For pioneer trees like *W. urens* , the positive association between *T*_t_ and lag time in the suboptimal and supraoptimal ranges might be relevant because the germination timing (early and fast germination) is crucial for successful establishment. Additionally, *T*_t_ might have higher predictive value than *T*_b_ and *T*_c_ for initiating, from seeds, greenhouse or field activities to initiate restoration, or to predict the effects of CC temperature changes on the future distribution of this species.

Thermal time separated two groups of seed populations: those produced from 1260 to 2040 m a.s.l., which exhibited higher slopes for suboptimal and supraoptimal thermal times fitted to linear models, compared to seeds produced from 2345 to 2500 m. Therefore, achieving full germination of seed populations may require more energy at higher elevations.

The cumulative temperature required to cover *T*_t50%_ (66.97°C day^−1^) was reached in three days for seeds collected at 2345 m a.s.l. (Figure 5). However, some authors have reported that seeds collected at the same elevation require a lag time of 3–10 days (González‐Zertuche et al. 2001; Gamboa‐deBuen et al. 2006), depending on the year of seed collection. Similarly, *T*_t50%_ was higher for *W. urens* seeds collected in 2013–2014 (68.75°C day^−1^; Martínez‐Villegas et al. 2018). Variations in temperature during seed development between years may explain differences in the time to reach 50% germination.

## 5 Conclusions

*Wigandia urens* seeds were collected along an elevational gradient that differed by 10.7°C ± 1.37°C between its elevational extremes, an amplitude higher than the increase in temperature (6°C) predicted by CC models for the next century.

*Wigandia urens* exhibited a wide plasticity; its germination was thermophilic. All five elevational seed populations germinated at > 30%–50% at 11°C–33°C, which might enable it to withstand thermal stress in afforested areas and inhabit temperate and tropical regions across a wide elevation gradient.

In an osmotic potential gradient, *W. urens* germinated at different percentages, which may allow it to respond with a staggered germination strategy to the erratic distribution of rain occurring in the REPSA at the beginning of the rainy season (> 40% of germination at −1 MPa) or to changes projected for precipitation due to CC.

Seed mass, maximum germination, and saturated:insaturated lipid ratios exhibited an increasing trend with elevation, which was disrupted by a drop after 2345 m a.s.l. At 2500 m, the limit of the elevational distribution of *W. urens* in Central Mexico, the lowest saturated:insaturated ratio was found.

*T*_b_ and *T*_c_ between elevational seed populations varied by 1°C–2°C; in contrast, *T*_t_ in the suboptimal and supraoptimal ranges showed significant differences between elevational populations. Thus, *T*_t_ might have a higher predictive value than *T*_b_ and *T*_c_ for initiating restoration activities from germination and for predicting the effects of CC temperature changes on the current and future distribution of this species.

The slopes for the linear relation between the percentile subpopulations and *T*_t_ separated two groups: one with the highest slopes (1260–2040 m) and the other with the lowest (2345–2500 m). The first group probably required less energy to reach the highest germination percentages.

At 2345 m, the cumulative temperature to reach *T*_t50%_ was covered in 3 days by seeds collected at this elevation. Nevertheless, based on the literature, lag time can be longer, depending on the year of seed collection.

There was a positive association between *T*_t_ and lag time, which, in the suboptimal temperature range, identified the *W. urens* germination timing (early and fast germination) required for the successful establishment of seasonal and pioneer species that need to be established early in the rainy season to avoid stressful factors during the dry season.

The germination traits, fatty acid profile, and their concentrations, along with the wide distribution of *W. urens* , identify it as a useful species for initiating a two‐step filter model in restoration ecology.

The ease of *W. urens* germination requirements favors its propagation in rural greenhouses using simple and cost‐effective technology.

## Author Contributions

**Ivonne Reyes‐Ortega:** conceptualization (lead), formal analysis (lead), investigation (lead), methodology (lead), validation (equal), writing – original draft (equal), writing – review and editing (equal). **María E. Sánchez‐Coronado:** conceptualization (equal), data curation (equal), formal analysis (lead), investigation (equal), methodology (supporting), validation (lead), visualization (lead), writing – original draft (lead), writing – review and editing (lead). **Susana Orozco‐Segovia:** conceptualization (equal), formal analysis (lead), supervision (lead), validation (lead), visualization (equal), writing – original draft (supporting), writing – review and editing (supporting). **César M. Flores‐Ortiz:** investigation (equal), methodology (equal), writing – original draft (equal), writing – review and editing (equal). **Gastón Contreras‐Jiménez:** formal analysis (equal), methodology (equal), validation (equal), writing – original draft (equal), writing – review and editing (equal). **Marco Solano‐De la Cruz:** formal analysis (equal), methodology (equal), validation (equal), writing – original draft (equal), writing – review and editing (equal). **Sonia Juárez‐Orozco:** formal analysis (equal), investigation (equal), methodology (equal), validation (equal), writing – original draft (equal), writing – review and editing (equal). **Zenón Cano‐Santana:** investigation (equal), validation (equal), writing – original draft (equal), writing – review and editing (equal). **Graciela García‐Guzmán:** investigation (equal), validation (equal), writing – original draft (equal), writing – review and editing (equal). **Jorge R. Blanco‐Martínez:** methodology (equal), validation (equal), writing – original draft (equal), writing – review and editing (equal). **Alma Orozco‐Segovia:** conceptualization (lead), data curation (equal), formal analysis (equal), funding acquisition (lead), investigation (lead), methodology (lead), project administration (lead), resources (lead), supervision (lead), validation (lead), visualization (lead), writing – original draft (lead), writing – review and editing (lead).

## Funding

This research was supported by Dirección General de Asuntos del Personal Académico, Universidad Nacional Autónoma de México (Grant PAPIIT IN‐204599, DGAPA), Consejo Nacional de Ciencia y Tecnología (Grant G0011‐N).

## Disclosure

Statement on inclusion: Our authors' inclusion considered Mexican researchers with a diversity of expertise and perspectives (from biochemistry to mathematical modeling), relevant for the development and analysis of this cutting‐edge study that addresses current and worldwide environmental problems such as climate change, which are also research priorities for Mexico, the country where the study was conducted. All authors equally contributed throughout various stages of the entire research process.

## Conflicts of Interest

The authors declare no conflicts of interest.

## Data Availability

This study's data is available at https://datosabiertos.unam.mx/IEUNAM:RESDATA‐IE:DEF_LEF_AO00001.

## Appendix 1

Results of the two‐way ANOVAs carried out to test the effect of the site of seed collection, germination temperature, and their interaction on the germination percentage or lag time of the seeds of *Wigandia urens* collected from five altitudinal populations of Central México and incubated in a temperature gradient.

**Table:**

Germination parameter	Source of variation	df	*F* ratio	*p*
Germination percentage	Elevational population (EP)	4224	157.53	> 0.0001
	Germination temperature (GT)	8224	7.06	> 0.0001
	EP × GT	32,224	1.55	> 0.039
Lag time (days)	EP	4224	15.97	> 0.0001
	GT	8224	6808.4	> 0.0001
	EP × GT	32,224	3.05	> 0.0001

## Appendix 2

Effect of the elevational population (EP), percentile subpopulation (PSp), and their interaction on the thermal requirements of the germination of *Wigandia urens* seeds from Central México. ns, non significant.

**Table:**

Temperature parameter	Source of variation	*F* ratio	df	*p*
Base temperature	EP (m a.s.l.)	9.00	4, 142	> 0.0001
	PSp (%)	5.84	5, 142	0.0001
	EP × PSp	0.87	20, 142	ns
Ceiling temperature	EP (m a.s.l.)	5.98	4, 142	0.0002
	PSp (%)	0.46	5, 142	ns
	EP × PSp	0.59	20, 142	ns
Suboptimal thermal time	EP (m a.s.l.)	60.73	4, 142	> 0.0001
	PSp (%)	62.16	5, 142	> 0.0001
	EP × PSp	1.08	20, 142	ns
Supraoptimal thermal time	EP (m a.s.l.)	42.60	4, 142	> 0.0001
	PSp (%)	41.00	5, 142	> 0.0001
	EP × PSp	0.74	20, 142	ns

## Appendix 3

### 3.1

Effect of the elevational population (EP), percentile subpopulation (PSp) and their interaction on the thermal requirements of the germination of *Wigandia urens* seeds from Central México.

**Table:**

Temperature parameter	Source of variation	*F* ratio	*d.f*.	*p*
Base temperature	EP (m a.s.l.)	9.00	4, 142	> 0.0001
	PSp (%)	5.84	5, 142	0.0001
	EP × PSp	0.87	20, 142	ns
Ceiling temperature	EP (m a.s.l.)	5.98	4, 142	0.0002
	PSp (%)	0.46	5, 142	ns
	EP × PSp	0.59	20, 142	ns
Suboptimal thermal time	EP (m a.s.l.)	60.73	4, 142	> 0.0001
	PSp (%)	62.16	5, 142	> 0.0001
	EP × PSp	1.08	20, 142	ns
Supraoptimal thermal time	EP (m a.s.l.)	42.60	4, 142	> 0.0001
	PSp (%)	41.00	5, 142	> 0.0001
	EP × PSp	0.74	20, 142	ns

Abbreviation: ns, non significant.
